# Bibliographical

**Published:** 1886-03

**Authors:** 


### EDITORIAL.

### Bibliographical.

Dental Medicine.—A Manuel of Dental Materia Medica and Therapeutics for Practitioners and Students, by Ferdinand J. S. Gorgas, A. M., M. D., D. D. S. &c., 80 pp. 379, 2d ed., Philadelphia, 1885. P. Blakeston, Son & Co.

We regret that we have not till now been able to give any extended notice to this excellent work. The necessity for the issue of a second edition is in itself the best proof of the general recognition of the value of the book, which, with the additional matter, may be fairly held to satisfy all the requirements of the dentist. We note especially the useful synopsis of treatment of affections of the oral cavity by prominent practitioners and the new therapeutic agents, to which might have been added iodol and acetate of alumina.

The growth of the materia medica is, however, not entirely a subject of congratulation. What is required is rather a profound study of the effects of the well known remedies rather than the continual addition of new names to the list, for it is quite apparent that by far the greater number of the remedial agents are mere names requiring, but in few cases only deserving, years of study and careful experiment before their true value can be determined. How little, for instance, is known of aconitine, even the variations of its comparative strength have received little, if any attention. In proof of this we note in the *American Druggist* extracts from a paper by Messrs. Buntzen and Madden, of Copenhagen, showing that the real study of the agent is only now being commenced.

The treatment of the subject of anasthetics is very full. We do not however, observe any reference to that most valuable mixture A. C. E., in which alcohol, chloroform, and ether are combined in the proportions of one, two, and three parts.

The typographical execution and general appearance of the book are excellent.

Chips From a Surgeon's Workshop ; No. VIII. Enamnelogenous Cysts. A. C. Bernays, 80 pp. 77, St. Louis.

This pamphlet adds to our knowledge of cystic tumors, some careful observations from which the author draws the conclusion that the formation of these epithelial multilocular cysts of the jaw is dependent upon the abnormal and heterotopic proliferation of the enamel germ or remnants of the enamel organ.

A list of works consulted is given. This might have been usefully increased by the addition of a reference to the dental journals in the volumes of which are many cases.

Scientific Adaptation of Artificial Dentures, by C. H. Land, 12 pp. 44, Detroit, 1885.

This book consists of two parts, the one treating of the subject of the teeth contains little that is new, except a harmless telt against received notions of atmospheric pressure, the other entitled useful hints for the laboratory, is, to judge from a protest at hand from Mr. Thomas Fletcher, entirely borrowed from his book, Practical Hints for the Laboratory and Operating Room, published in 1873. Of the two parts, the latter is really valuable, and had Mr. Land seen fit to acknowledge its alleged source, the thanks of the profession would have been due to him for its republication.

The art of making and printing books is in these later days becoming a little too easy.

